# The Pathobiology of the Meniscus: A Comparison Between the Human and Dog

**DOI:** 10.3389/fvets.2018.00073

**Published:** 2018-04-16

**Authors:** Olga Krupkova, Lucas Smolders, Karin Wuertz-Kozak, James Cook, Antonio Pozzi

**Affiliations:** ^1^Small Animals Surgery, Tierspital, Zurich, Switzerland; ^2^Department of Health Sciences and Technology, Institute for Biomechanics, ETH Zurich, Zurich, Switzerland; ^3^Spine Center, Schön Klinik München Harlaching, Munich, Germany; ^4^Academic Teaching Hospital and Spine Research Institute, Paracelsus Private Medical University Salzburg, Salzburg, Austria; ^5^Department of Health Sciences, University of Potsdam, Potsdam, Germany; ^6^Missouri Orthopaedic Institute, University of Missouri, Columbia, SC, United States

**Keywords:** meniscus, inflammation, oxidative stress, pain, dog

## Abstract

Serious knee pain and related disability have an annual prevalence of approximately 25% on those over the age of 55 years. As curative treatments for the common knee problems are not available to date, knee pathologies typically progress and often lead to osteoarthritis (OA). While the roles that the meniscus plays in knee biomechanics are well characterized, biological mechanisms underlying meniscus pathophysiology and roles in knee pain and OA progression are not fully clear. Experimental treatments for knee disorders that are successful in animal models often produce unsatisfactory results in humans due to species differences or the inability to fully replicate disease progression in experimental animals. The use of animals with spontaneous knee pathologies, such as dogs, can significantly help addressing this issue. As microscopic and macroscopic anatomy of the canine and human menisci are similar, spontaneous meniscal pathologies in canine patients are thought to be highly relevant for translational medicine. However, it is not clear whether the biomolecular mechanisms of pain, degradation of extracellular matrix, and inflammatory responses are species dependent. The aims of this review are (1) to provide an overview of the anatomy, physiology, and pathology of the human and canine meniscus, (2) to compare the known signaling pathways involved in spontaneous meniscus pathology between both species, and (3) to assess the relevance of dogs with spontaneous meniscal pathology as a translational model. Understanding these mechanisms in human and canine meniscus can help to advance diagnostic and therapeutic strategies for painful knee disorders and improve clinical decision making.

## Introduction

The knee is one of the joints most commonly affected by osteoarthritis (OA), usually secondary to anterior cruciate ligament (ACL), meniscal injuries, trauma, or overuse (1). Approximately 25% of people over the age of 55 have suffered from a significant episode of knee pain in the past year of their life, with half of these people reporting associated disability (1). As curative treatments for these common knee problems are not available to date, the diseases typically progresses, often leading to OA and the associated chronic pain and disability. With aging of the population and increasing obesity, the prevalence and socioeconomic impact of painful knee pathologies are expected to rise (1, 2).

The knee joint is composed of the fibrous joint capsule, the synovial membrane, the joint cavity with synovial fluid (SF), menisci, ligaments, and bones lined with articular cartilage. The overall function of the knee joint is to allow motion and provide stability for load transfer between femur and tibia. Anatomy and function of the knee undergo age-related changes caused by shifts in cell density and phenotypes and molecular, structural, and mechanical alterations of the extracellular matrix (ECM), influencing the load distribution and joint kinematics. Damage to any of the knee structures can lead to loss of biological and biomechanical homeostasis and contribute to progressive degeneration of the whole joint. Despite the clinical importance of pain associated with degenerative joint disease, it is still unclear which structures of the knee are specifically painful and to what extent. While the meniscus plays a crucial role in biomechanics of the knee, its role in pain generation is not fully clear. Explaining the biological mechanisms underlying meniscus pathophysiology can therefore help to advance therapeutic strategies against progression of painful knee disorders.

Limitations to effective clinical application of translational joint research can be caused not only by species-related differences in metabolism, biology, and biomechanics but also by the inability to fully replicate the course of spontaneous pathology in animals. Pre-existing pathophysiological mechanisms underlying progressive knee degeneration in aging humans are commonly not active in experimental animals (3), causing overestimation of therapeutic effects (3, 4). As an example, human knees often have been under the influence of altered mechanical loading, catabolism, and inflammation for a long time before becoming symptomatic. Therefore, it can be expected that the healing responses of the symptomatic human knee are diminished when compared with joints of experimental animals that are younger and/or treated at or near the time of insult. The use of animal models with spontaneous joint pathology can significantly help address this limitation to effective translational joint research.

Both microscopic and macroscopic anatomy of the canine and human menisci are similar, however, biomolecular differences underlying meniscus pathologies in human and dog, have not yet been fully elucidated. Therefore, the aims of this review are (1) to provide an overview of the anatomy, physiology, and pathology of the human and canine meniscus, (2) to compare the known signaling pathways involved in spontaneous meniscus pathology between both species, and (3) to assess the relevance of dogs with spontaneous meniscal pathology as effective translational models for clinical application to human meniscal pathology.

## Human Meniscus

### Anatomy and Physiology

The medial and lateral menisci are semicircular fibrocartilages anchored to the tibia and femur by meniscal ligaments. Although the gross anatomy of human and canine menisci is similar, small differences can be found in the meniscal attachments to femur and tibia (5).

The contributions of the menisci to knee joint function include absorption and distribution of mechanical loads, congruity and stabilization, lubrication, nutrition, and mechano- and proprioception (6). The meniscal tissue is composed of water (±70%), collagens (±20%), glycosaminoglycans (GAG) (±1%), non-collagenous proteins (1%), and cells (7, 8). Collagen type I is a major component of the ECM, which is produced by the cells. Collagen type I is distributed throughout the whole meniscus, from the peripheral to inner area, organized in circumferential fibers to culminate in the anterior (cranial) and posterior (caudal) menisco-tibial ligaments (7, 9, 10). Collagen type II is found mainly in the inner avascular zone, where it shows an organized network of circumferential and radial fibers (7, 10). An organized network of proteoglycans (aggrecan, decorin, and biglycan) is also predominantly located in inner zone (7, 11). Adhesion glycoproteins such as fibronectin and thrombospondin connect cells with the surrounding ECM (8). No major differences in composition are found between human and canine meniscus (4).

Cells found in human and canine menisci are classified as chondrocytes and fibrochondrocytes, distributed according to the ECM they produce: chondrocyte-like cells are located in inner (avascular) part of the menisci, particularly in the anterior and posterior horns, whereas fibrochondrocytes are found in the outer (vascular) layers (7, 8). Meniscal cells are involved in responses to mechanical loading, osmolarity, and pressure (12). Mechanical loading is crucial for the health and function of the meniscus, as it can drive either anti-inflammatory or pro-inflammatory responses as well as influence the balance of ECM turnover toward anabolism or catabolism, depending on magnitude, frequency, and duration, in both humans and dogs (12, 13). Mechanisms involved in mechanosensing and osmosensing of meniscal fibrochondrocytes are not fully understood. Nevertheless, cell substrate-mediated responses, mainly driven by calcium, have been identified *in vitro* (14–16). Meniscal cells also contribute to the joint lubrication by secreting mucoproteins into the SF (17), produced by penetration of a plasma filtrate from vascularized synovial membrane. Apart from plasma proteins, SF also contains molecules secreted by articular chondrocytes and synovial cells, including hyaluronan and lubricin, respectively. The function of the SF is to facilitate joint movement, absorb mechanical loads, and provide transport medium for exchange of gases, nutrients, and waste products. Importantly, the SF in injured and degenerative joints contains pro-inflammatory cytokines, catabolic enzymes, and pain mediators, spreading them to non-affected parts of the joint and promoting disease progression and pain (17).

In both human and dog, approximately 25% of the meniscus (outer part) is vascularized, while the inner part receives nutrition by diffusion from the SF (18, 19). Therefore, the outer zone has higher capacity to heal spontaneously, while inner meniscus has much more limited healing capacity. Healing mechanisms in the vascularized zone include cell-mediated tissue repair (by stem cells, neutrophils, macrophages, and lymphocytes), tissue-remodeling molecules, oxygen, and nutrients. As the inner meniscus is not connected to the bloodstream, inner meniscal tears have little capacity to heal, typically resulting in maceration and degeneration of the affected meniscal tissue (7). Innervation of the meniscus coincides with the vascularization pattern, as most nerves are associated with vessels. The outer one-third of the meniscus and the anterior and posterior horns are innervated by nerves providing proprioceptive and sensory function (6). Mechanoreceptors are located at the horns and attachment structures, whereas free nerve endings are found throughout the meniscus, except for the inner one-third of the meniscal body (20).

### Pathology

Meniscal lesions represent the most common intra-articular knee injury and are the most frequent cause for knee surgery in humans (7, 21). The younger population typically suffers from traumatic meniscal injuries (e.g., due to sports) with or without associated ligament ruptures, while older people are affected by degenerative tears that can be symptomatic or asymptomatic (8, 22). Importantly, meniscal damage is associated with main painful knee pathologies both in human and dog (6, 23, 24). Common human knee pathologies are described below. Although less is known about underlying pathophysiological mechanisms on canine stifle disorders, these mechanisms are thought to be similar.

#### Aging of the Knee Joint

The prevalence of knee pain increases with age (1). The normal aging process is caused by a progressive loss of cell function and ability to effectively maintain the ECM. Therefore, age-related changes in menisci and cartilage of both *human* and *dog* arise from natural senescence process (25, 26). The effects of aging on meniscus in *human* include loss of collagen fiber organization, decreased cell function, and reduced cell density, loss of water content, and associated detrimental changes to its material properties (25, 27). Anisotropies in the ECM give rise to variations in the distribution of local stress and strain and alter cell and ECM responses to mechanical loading (27, 28). Structural disorganization of the ECM can progress to meniscal lesions (29). Physiological loading has beneficial effects on the aging meniscus by promoting transport of nutrients through the inner avascular part. However, the meniscal repair capacity is reduced with age such that even physiological loading can result in pathology (26, 30).

#### Cruciate Ligament Ruptures

Traumatic ligament ruptures are common in athletes and physically active *people*, while ligament degeneration associated with OA affects the elderly (31). Ligament ruptures alter joint kinematics and cause instability and abnormal loading, contact areas, and contact pressures. As a consequence of abnormal knee biomechanics, the meniscus is at higher risk for impingement and damage. ACL ruptures and meniscal tears eventually result in OA (23). It has been suggested that up to 80% of knees with an injured ACL demonstrate evidence of OA and meniscal damage at 5–15 years after initial injury (32). Age-related decline in ligament structure includes ECM degeneration and shifts in cell density and phenotypes both in *human* and *dog* (33, 34).

#### Osteoarthritis

Osteoarthritis, the most prevalent form of skeletal disease, represents a leading cause of disability in middle and old age. OA affects 40% of *people* above 70 years of age, being more prevalent than any other form of arthritis (35). OA is associated with a progressive loss of articular cartilage; however, the details of its etiology and pathogenesis remain unclear. OA knees are characterized by degenerative destruction of articular cartilage, meniscal tears and degeneration, and changes in ligament integrity accompanied by pain. Due to the complex nature of the knee joint, OA can be both a consequence and a cause of meniscal tears (23). A spontaneous or traumatic meniscal tear can disturb knee biomechanics, leading to OA, but knee OA can also cause a meniscal tear by abnormal loading and breakdown of meniscal structure (22). Meniscal vascular densities are increased in human patients with OA. However, the contribution of meniscal angiogenesis to symptom progression and joint pathology in OA is unclear (36).

Normal aging process and degeneration of menisci can be accelerated by several risk factors, such as gene polymorphisms. A polymorphic gene consists of an allele that causes abnormal gene expression and protein production, influencing the onset and severity of associated disorders (37). Several studies identified gene polymorphisms that can significantly influence the pathology of the knee and stifle of humans and dogs, respectively. Gene polymorphisms can alter promoter activity, influence transcription factor binding, and may result in shorter transcripts or faster product decay. Investigating polymorphisms associated with meniscus pathology could provide insight into disease mechanisms, reveal therapeutic targets, and help identify patients at high risk. However, apart from two studies that reported an association of polymorphisms in growth differentiation factor 5 (*GDF5*) and CD40 with meniscus injury in Chinese population (38, 39), polymorphisms associated with meniscal injury are still unknown. Targeting molecular pathways regulated by polymorphic genes may become important in the development of patient-specific treatments. Other risk factors that contribute to the progression of meniscal pathology include systemic inflammation, bacterial infection, overloading (e.g., due to overweight), and injury (23, 40, 41).

## Translational Models for Meniscal Pathology

Animal models are crucial in facilitating translation of basic research findings to practical clinical applications that ultimately enhance human health and quality of life. Biological mechanisms of meniscal damage and repair have been tested both in small and large animal models (7, 42, 43). In general, large animal models appear more suitable for meniscal biological studies than small animals. Large animals such as sheep or dogs possess a joint anatomy and tissue composition close to humans. Their tissue characteristics include comparable molecular diffusion distances and biomechanical behavior, making large animals more relevant for translational therapeutic testing. As an example, sheep have meniscal dimensions, vascularization pattern, cellularity, and collagen structure similar to humans (7, 9, 44). However, large animal models require higher costs, especially if studies are focused on long-term effects of experimental treatments. Small animals such as rodents and rabbits can be successfully used for uncovering biological mechanisms underlying tissue functions as well as for pilot therapeutic studies. However, their distinct anatomy and physiology make them less relevant for translational therapeutic testing. As an example, rabbit menisci contain significantly more cells, more expansive vascularization pattern, and different collagen structure compared with humans (7, 9, 45). Therefore, the choice of animal to be used to test a given hypothesis depends on several factors including study design, length, budget, outcome measures, stage of development, and intended application (46, 47). Detailed reviews on translational animal models for degenerative disorders of the knee are available (4, 46).

To date, most studies aimed at translational meniscal research have focused on simulating the effects of partial or total meniscectomies in animal models, with the goal of studying the roles of the deficient meniscus in the development of OA and methods to mitigate associated joint pathology (7). However, results obtained from these studies cannot fully assess the effects of degenerative or traumatic meniscal tears in the development and progression of OA. Animal models where meniscal injury is induced by transection of the cranial cruciate ligament (CCL), equivalent to ACL, allow for study of mechanical and degenerative meniscal pathology associated with instability, but are also limited in clinical applicability based on untreated instability and the inability to mimic age-related degenerative changes in the knee.

The most frequently used large animal model for knee pathologies is the dog (47). The canine stifle has similar anatomical and physiological characteristics to the human knee (4, 48, 49). The canine meniscus has comparable vascularization pattern, cellularity, collagen structure, and similar permeability (7, 45). However, dog’s menisci differ from human menisci in biomechanical properties such as aggregate modulus, shear modulus and anatomy, indicating that caution is needed when choosing the dog as a model for biomechanical studies (5, 45). Two types of canine meniscal pathology can be distinguished: experimentally induced and spontaneous.

### Experimentally Induced Meniscal Pathology in Dog

Experimentally induced meniscal pathology is created using several canine models designed to study meniscal deficiency and associated OA, as well as meniscal repair and regeneration treatment strategies. In these models, summarized in Table 1, meniscal pathology is either surgically created (meniscectomy, meniscal release) or indirectly induced with CCL transection. As outlined above, each of these models have limitations with respect to clinical applicability. For this reason, accurate modeling of meniscal degeneration may require study of spontaneously occurring meniscal pathology (47).

**Table 1:** Characteristics of experimental canine models for meniscal pathologies.

Model	Trigger	Meniscal damage	Resulting OA	Synovial inflammation	Reference
Cranial cruciate ligament/anterior cruciate ligament transection	Joint instability	Secondary	Moderate to severe	Yes	(47, 50)
Meniscal release	Joint instability	Primary	Moderate to severe	Yes	(51–53)
Meniscectomy	Joint instability	Removal	Related to the size of the preserved meniscus	Yes	(7, 54)
Cartilage grooving	Chondral damage	Secondary	Mild	Yes	(47)

### Spontaneously Occurring Meniscal Pathology in Dog

**Figure 1:**
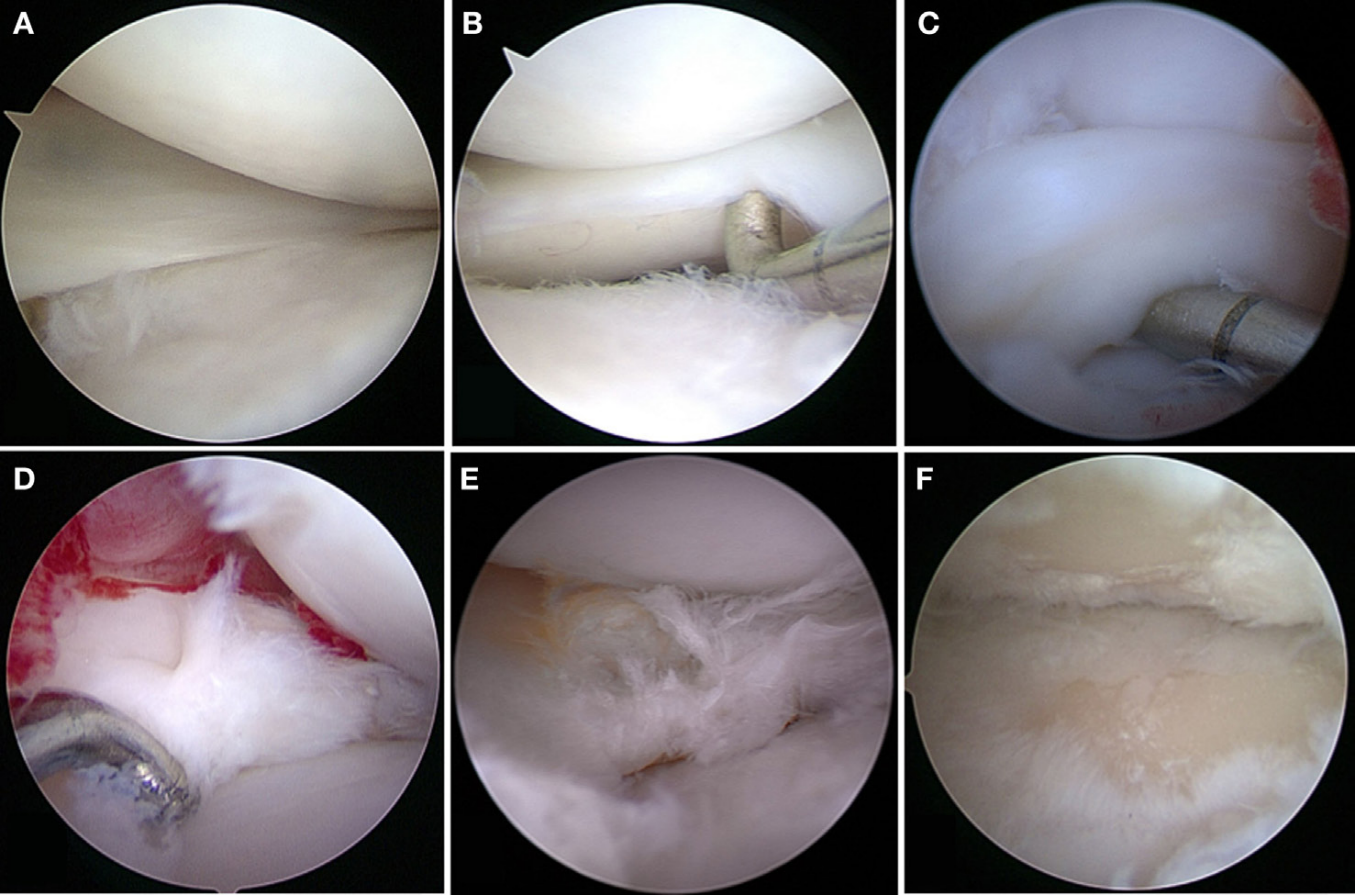
Meniscal tears in dogs. **(A)** Grossly normal meniscus with outerbridge grade 1 cartilage pathology; **(B)** grossly normal meniscus with outerbridge grade 2 cartilage pathology; **(C)** displaced bucket handle tear; **(D)** acute complex tears without gross evidence of degeneration; **(E)** complex tears with gross evidence of degeneration; **(F)** outerbridge grade 4 of both femoral and tibial articular surface associated with meniscal tear.

**Figure 2:**
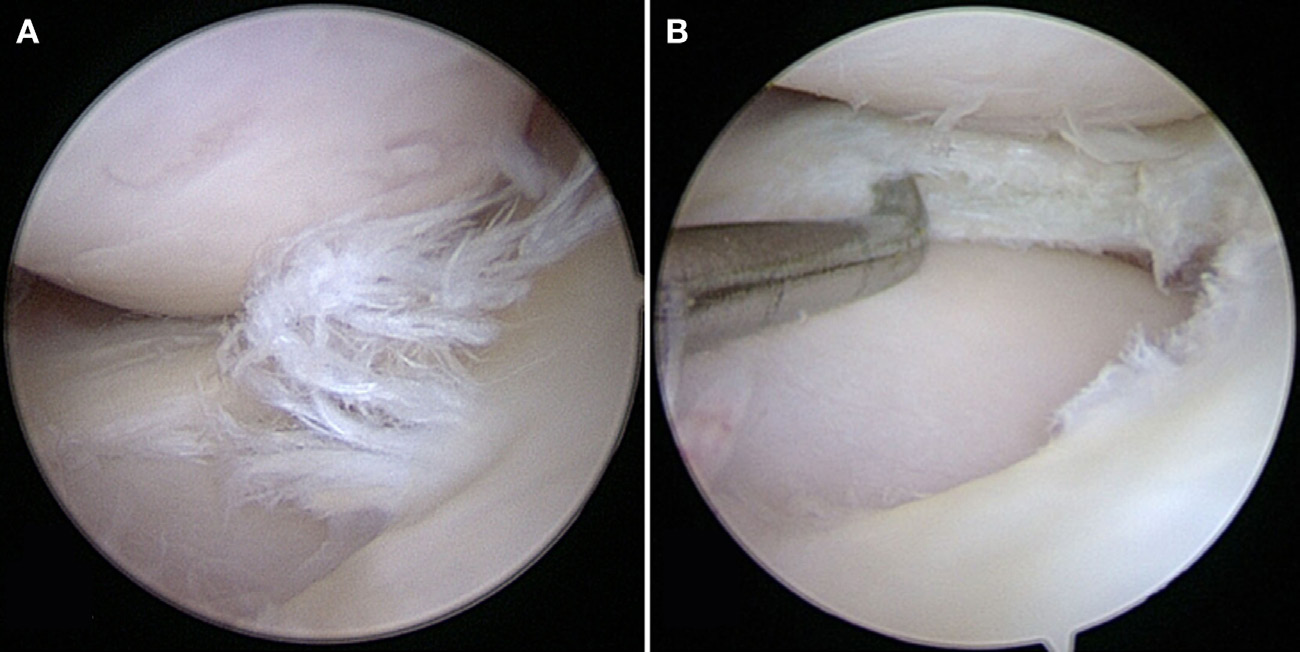
**(A)** A degenerative meniscal tear characterized by fibrillation, softening, and discoloration. **(B)** An horizontal cleavage tear is apparent when probing the mid-substance of the meniscus after performing a partial meniscectomy.

Although experimentally induced pathologies have certain advantages, such as reduced disease variability among subjects, spontaneous meniscal pathologies in canine patients are highly relevant for translational medicine, especially when analyzing degenerative processes. Spontaneously occurring meniscal pathology can be well studied in dogs, as they are prone to CCL disease with concurrent or subsequent meniscal tears and secondary OA (6, 49) (Figures 1 and 2). Canine CCL ruptures occur most commonly due to a chronic degenerative weakening of the ligament causing premature failure and joint instability (6, 55, 56). Consequently, femoro-tibial subluxation causes shearing forces on the meniscus, which ultimately fails presumably adding to pain and joint inflammation. Another common type of meniscal pathology in dogs is the degenerative tear (57). These tears can present with fraying of the free edge, centrally located horizontal tears, fringe tags, interstitial tears, radial tears, and/or extensive fibrillation of the meniscus. These meniscal lesions are almost invariably accompanied by articular cartilage lesions ranging from softening to full-thickness defects and associated subchondral bone changes. Both traumatic and degenerative meniscal tears are main causes of degenerative joint disease in canine patients (17, 23). It has been estimated that approximately 20% of dogs older than 1 year of age are affected by OA (49). Similarities between canine and human patients are not only related to disease progression but also to environmental factors (58).

Dogs with spontaneously occurring meniscus disorders are useful for studying the roles of meniscus in stifle/knee pathologies and for therapeutic testing. Another important advantage of canine patients is the possibility to assess pain *via* direct observation focused on the degree of lameness, gait analysis, and subjective rating scales. Subjective rating scales range from single visual analog scale assessments to extensive questionnaires typically completed by the animal’s owners. The questionnaires, comparable to questionnaires used in human clinical trials, assess pain through its impact on health-related quality of life. Although quality of life and reduction of pain are important endpoints in human clinical trials, similar pain scoring systems do not exist for other large animal species (59). Apart from the scoring systems, biomarkers can be significant diagnostic tools to assess progression of the disease and the level of pain. OA biomarkers that may possibly differentiate between spontaneous CCL disease and other types of OA in canine patients have been identified (60).

Furthermore, diagnostic imaging, arthroscopic interventions, and postoperative management similar to human medical practice are routinely used in veterinary medicine (4). Depending on a study’s design and objectives—especially in the context of translation of meniscal therapies—certain similarities and differences between human and canine meniscal pathophysiology and biological mechanisms underlying progression of meniscal degeneration and related pain may be of specific importance and will hence be discussed in more detail in the following sections.

## Biological Hallmarks of Meniscal Pathology in Human and Dog

**Figure 3:**
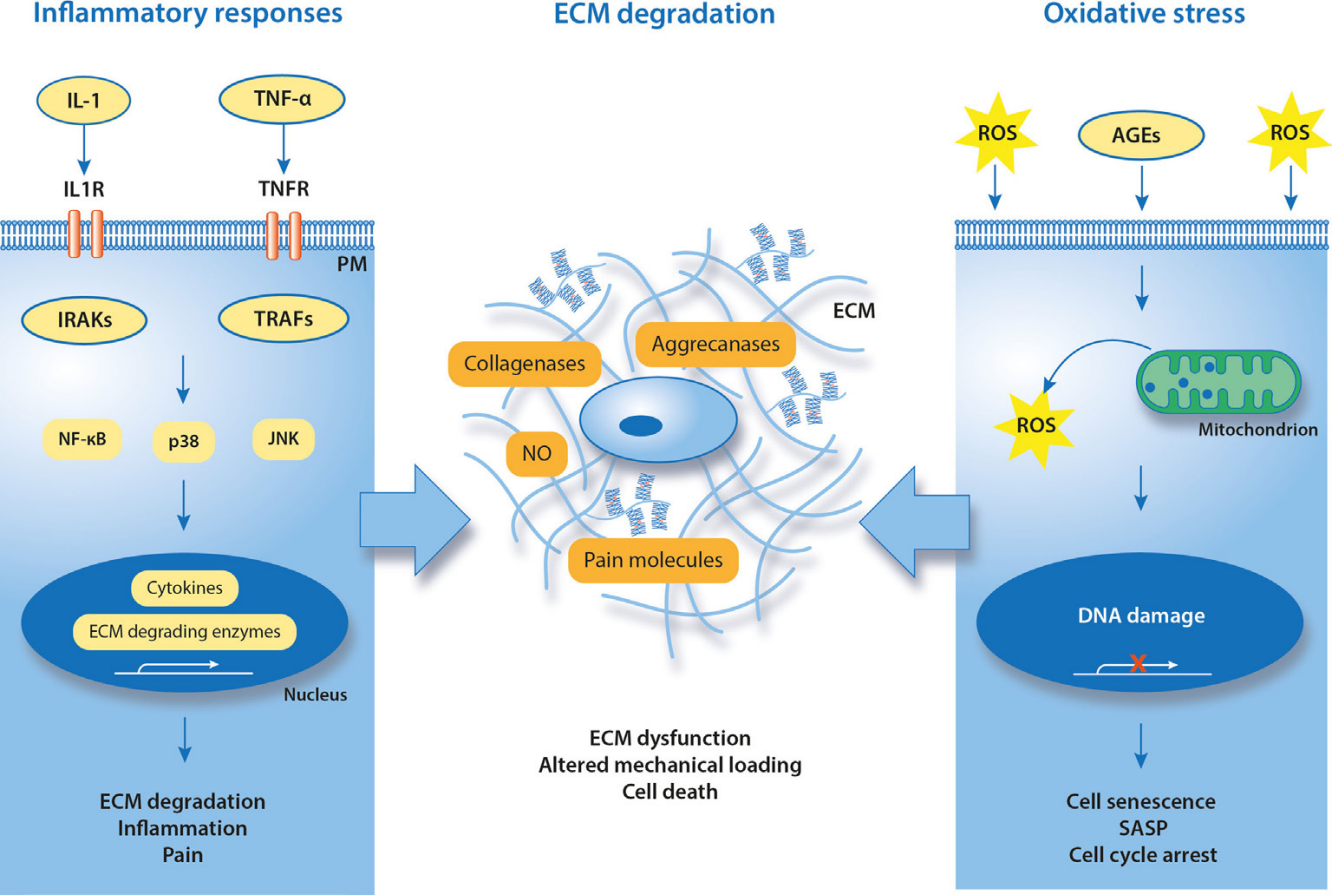
Known and putative molecular hallmarks of painful meniscal pathology. Suggested mechanisms in meniscal pathophysiology are inflammation and oxidative stress that together contribute to degradation of ECM. IL-1, interleukin-1; TNF-α, tumor necrosis factor alpha; IL1R, interleukin-1 receptor; TNFR, TNF receptor; TRAF, TNF receptor-associated factor; IRAK, interleukin receptor-associated kinase; ROS, reactive oxygen species; NO, nitric oxide; SASP, senescence-associated secretory phenotype; AGEs, advanced glycation end products; PM, plasma membrane; ECM, extracellular matrix.

Progression of knee pathology is a result of both chemical and mechanical perturbations arising from complex interactions between all tissues of the knee. As one of these tissues, the meniscus can have active roles in disease progression and pain. Typical biological hallmarks that are seen during progression of knee disorders are inflammation, ECM degradation, and oxidative damage. Molecules and pathways involved in both age-related and trauma-induced damage of the meniscus are illustrated below and summarized in Table 2 and Figure 3.

**Table 2:** Possible therapeutic targets in menisci and SF associated with aging and degenerative knee/stifle disorders.

Therapeutic targets		Type of dysregulation	Found in human	Found in dog
Extracellular matrix (ECM) constituents	Glycosaminoglycans	Degradation	M: unchanged SF: increased (61)	M: unchanged (62) SF: fragments (63, 64)

	Collagens	Degradation	M: reduced (27, 65) SF: increased (66)	M: uk SF: uk

ECM remodeling	ADAMTS4	Overexpression/activity	M: yes (67) SF: (68, 69)	M: uk SF: uk

	ADAMTS5	Overexpression/activity	M: uk SF: (68)	M: uk SF: uk

	MMP1	Overexpression/activity	M: yes (67) SF: yes (70)	M: uk SF: yes (71)

	MMP2	Overexpression/activity	M: yes (67) SF: yes (72)	M: uk SF: yes (73)

	MMP3	Overexpression/activity	M: yes (67) SF: yes (70)	M: uk SF: yes (63) no (74)

	MMP8	Overexpression/activity	M: yes (67) SF: yes (70)	M: uk SF: uk

	MMP9	Overexpression/activity	M: yes (75) SF: yes (72)	M: uk SF: yes (74)

	MMP13	Overexpression/activity	M: yes (67) SF: yes (76)	M: uk SF: no (74)

Inflammation	IL-1α	Overexpression/activity	M: yes (67) SF: yes (77)	M: uk SF: uk

	IL-1β	Overexpression/activity	M: yes (67) SF: yes (77)	M: uk SF: yes (63)

	IL-6	Overexpression/activity	M: yes (67) SF: yes (77)	M: uk SF: yes (63, 78)

	IL-8	Overexpression/activity	M: yes (67) SF: yes (77)	M: uk SF: yes (79)

	TNF-α	Overexpression/activity	M: yes (67) SF: yes (77)	M: uk SF: yes (63)

	14-3-3	Overexpression/activity	M: uk SF: yes (80)	M: uk SF: yes (81)

Pain	Nerve growth factor	Increase	M: uk SF: yes (82)	M: uk SF: yes (83)

	Adenosine triphosphate	Increase	M: uk SF: (84)	M: uk SF: yes (85)

	Cyclooxygenase-2, prostaglandin E2	Overexpression/activity	M: yes (86, 87) SF: yes (88)	M: uk SF: (89)

	Substance P	Increase	M: uk SF: yes (90)	M: uk SF: uk

	Calcitonin gene-related peptide	Increase	M: yes (91) SF: yes (92)	M: uk SF: (58)

	Lactic acid	Increase	M: uk SF: uk	M: uk SF: uk

Oxidative stress	Advanced glycation end product/receptor for advanced glycation end products	Increase overexpression/activity	M: yes (87) SF: yes (93, 94)	M: uk SF: uk

	Reactive oxygen species, NO	Increase	M: yes (86, 95) SF: yes (96)	M: uk SF: yes (82, 97)

*M, meniscus; SF, synovial fluid; uk, unknown*.

### Inflammatory Responses

It has been confirmed that inflammation is a key factor in progression of many of the human knee disorders leading to OA (98). Although articular chondrocytes and cells of the synovial membrane are main contributors to the inflammatory and catabolic reactions of the knee, cells of ligaments and menisci also overexpress pro-inflammatory and catabolic mediators. In addition, pro-inflammatory cytokines can be produced by immune cells that infiltrate joints due to trauma or age-related cartilage degeneration. Severe inflammatory responses in synovium and synovial membrane are characteristic for immune-mediated disorders (e.g., rheumatoid arthritis), but pro-inflammatory cytokines are also active in OA joints (99). In both cases, cytokines stimulate cells of neighboring tissues (chondrocytes, ligaments, and menisci), contributing to disease progression. The SF plays an important role as it spreads inflammatory and catabolic mediators within the knee joint.

Interleukin-1 (IL-1α, IL-1β) and tumor necrosis factor alpha (TNF-α) are consistently found in degenerative joints. IL-1β and TNF-α can stimulate chondrocytes to induce the expression of collagenases (MMPs) and aggrecanases (ADAMTSs), promoting degradation of the articular tissues’ ECM. At the same time, IL-1β and TNF-α activate the expression of other pro-inflammatory and pain-generating mediators (100, 101). General mechanisms for IL-1β and TNF-α action in chondrocytes is *via* cell surface receptors. Through its receptor IL-1R1, IL-1β can activate nuclear factor-κB (NF-κB), p38 mitogen-activated protein kinase (MAPK), and/or c-Jun N-terminal protein kinase (JNK). The activation of these signaling molecules results in transcription and translation of genes for inflammatory cytokines, ECM-degrading enzymes, and molecules that promote pain. The interaction of TNF-α with its receptor TNFR1 also can activate NF-κB and MAPK pathways, through tumor necrosis factor receptor-associated factors (67).

Meniscal cells and tissues can be stimulated by pro-inflammatory cytokines, providing a good model to study involvement of inflamed meniscus in disease progression as well as for therapeutic testing. It has been reported that primary cells derived from degenerative *human* menisci and stimulated with pro-inflammatory factors (e.g., IL-1β) exhibit similar inflammatory and catabolic responses as chondrocytes. These cells overexpressed ECM-degrading enzymes (MMP1, MMP3, MMP13, andADAMTS4), cytokines (IL-1α, IL-1β, and IL-6), chemokines (IL-8, CXCL1, CXCL2, and CSF1), and components of the NF-κB pathway, and underexpressed collagen (67). In human meniscal explants, IL-1α and IL-1β impair intrinsic repair capacity by increasing expression of MMPs. In addition, MMP inhibitors can reduce MMP activity and enhance repair in meniscal explants, confirming the importance of MMPs in meniscal homeostasis (100, 101). Similar data were obtained in *porcine* and *ovine* meniscal *in vitro* models (102). These studies all together suggest that meniscal cells are responsive to inflammatory stimulation and thus can contribute to progression of knee degeneration and pain. However, inflammatory mechanisms in canine meniscus have not yet been thoroughly investigated.

Although inflammation-related cell and tissue culture studies with canine menisci are rare, the inflammatory profile of the painful *canine* stifle has previously been studied in canine patients and animal models. Similar to humans, the SF of dogs with CCL transection-induced OA and naturally occurring CCL ruptures contains increased levels of IL-1β, IL-6, IL-8, TNF-α, MMP1, MMP3, MMP9, C-reactive protein, and 14-3-3-g, compared with healthy SF (58, 63, 78, 79, 81, 103, 104). Although the role of 14-3-3 proteins in joint inflammation is not yet established, it was shown in humans that these proteins control cell cycle, cell growth, differentiation, survival, apoptosis, migration, and spreading (80, 105). SF of the canine stifle also contains adenosine triphosphate (ATP), with a possible role in pain sensation (85). A correlation between the concentration of SF ATP and OA knee pain has previously been demonstrated in humans (84), but not yet in dogs. Apart from SF, elevated IL-1β, IL-6, and IL-10 were found in the synovial membrane of dogs with both OA and CCL ruptures (106). In addition, inflammation of SF is associated with depletion of proteoglycans from articular cartilage and possibly also from menisci. The activity of IL-1β, IL-6, and MMP3 in SF of canine stifle joints with naturally acquired CCL ruptures correlates with GAG content in the SF, but the source of GAG has not been determined (63, 107). Synovium and SF-related inflammatory responses in humans do not only occur in OA and ligament ruptures but also after meniscal injuries (108). However, the association between canine meniscal pathology, SF inflammation, and inflammatory responses of meniscal cells remains largely unknown.

Aside from chondrocytes, infiltrating immune cells can be another source of inflammation, although their roles in meniscal pathology and OA are unclear. Macrophages, T-cells, mast cells, B-cells, and plasma cells can be found in *human* and *canine* OA synovium, where they release inflammatory cytokines into SF (109, 110). The synovial membrane and the CCL of dogs with CCL ruptures contain significantly increased levels of antibodies to type I and type II collagen, IgG, and IgM, and increased expression of intercellular cell adhesion molecule-1 and IL-8, when compared with healthy controls (55, 103, 111). Canine OA SF contains macrophage-like cells that express significantly more collagenolytic enzymes, tartrate-resistant acid phosphatase (TRAP), and cathepsin K compared with healthy SF (104). Importantly, synovial macrophage-like cells that produce TRAP and cathepsin K are significant features of the CCL ruptures in dogs, but not in humans, suggesting species-specific differences in immune responses associated with cruciate ligament ruptures (33). Although these findings provide first evidence on the involvement of immune cells in inflammation and cartilage destruction, exact mechanisms and relative contribution to OA remain unknown in both species. Furthermore, acute immune reactions are considered beneficial for joint healing. Therefore, a complete inhibition of these reactions may not be beneficial.

In addition to the catabolic and pro-inflammatory effects, secreted inflammatory cytokines are also involved in the neuronal sensitization and transduction of pain as well as in controlling the rate of cell senescence. Human and canine joint disease progresses due to the contributing activities of pro-inflammatory and catabolic signaling pathways. Nevertheless, significance and spatiotemporal expression and activity of individual molecules still have to be elucidated.

### ECM Changes

Healthy human and canine menisci contain predominantly collagen type I produced by fibrochondrocytes (27). Collagen is synthesized as pro-collagen and secreted into the ECM, where final collagen assembly takes place. Flexible collagen fibers are cross-linked by lysyl oxidases to gain additional strength. Under physiological conditions, collagen is stable (112). Menisci also contain proteoglycans (aggrecan, versican, and perlecan) in lower quantities.

Degradation and lamellar disorganization of collagen fibers occurs in human aging and contributes to likelihood of meniscal tears. Meniscal tears alter the stress distribution in the meniscus, increasing the risk for load-induced knee injuries (12). Mechanical loads also influence ECM turnover, as non-physiological loading of the meniscus can induce synthesis of catabolic and pro-inflammatory genes and reduce cell viability (12, 96). On the other hand, physiological loading promotes ECM turnover in both articular cartilage and menisci. Physiological loading regimes were shown to inhibit inflammatory and catabolic responses and activate anabolic genes in *human* articular chondrocytes (28) and induce production of collagens and meniscal repair in a *canine* model (13). It was also demonstrated that physical activity modulates the synthesis of collagens in the hyaline cartilage of aging dogs, partially preserving the cartilage structure (26). Molecular mechanisms behind responses to mechanical loading in meniscal and articular chondrocytes remain largely unexplored, due to the inhomogeneous composition and the anisotropic structure of these tissues. Intrinsic coupling of tensile, compressive, and shear stresses *in vivo*, as well as complex osmolarity and fluid flow environments complicate the design of experiments (12). Nevertheless, it has been shown that cells in the middle and inner *human* (and ovine) meniscus contain a pericellular region, with a possible role in shielding these cells from large mechanical strains and stresses. The pericellular region is formed mainly by the proteoglycan perlecan that connects cell surface molecules with ECM components (113). Interestingly, the perlecan content in menisci is reduced with age (114). However, the existence and importance of meniscal pericellular region in dogs remains unknown.

Collagen can be cleaved enzymatically by collagenases (MMPs), whereas proteoglycans are degraded by aggrecanases (ADAMTS), expressed by meniscal fibrochondrocytes and other cell types such as synovial cells and articular chondrocytes. Balanced expression of MMPs and ADAMTS ensures normal ECM turnover in a healthy joint. However, the ability of human cartilaginous tissues to maintain the ECM declines with age and with the degree of degeneration (26, 27, 107). Overexpressed collagenases and aggrecanases are released within the knee joint and transported by the SF, enhancing the local catabolic environment. Elevated MMPs were also found in the SF of *canine* patients with CCL ruptures and OA (63, 104), but their expression/activity in canine menisci was not yet studied. It is likely that an increased activity of canine ECM-degrading enzymes, together with age-related collagen dysfunction and mechanical overloading, can reduce exchange of solutes structure, impairing repair capacity of the meniscal ECM, and promoting degeneration. Apart from collagenases and aggrecanases, *human* ECM of cartilaginous tissues contains antagonists of inflammatory pathways (e.g., interleukin-1 receptor antagonist), enzyme inhibitors and activators, and other molecules diversely affecting cell fate. The importance of these molecules in canine menisci remains to be elucidated.

Cells can sense their local environment *via* cytoskeleton and membrane receptors (115). Ion channels on cell surfaces can be activated mechanically, by deformations of the ECM that pull or push on these receptors, or chemically, by changes in concentrations and activities of their ligands. Opening ion channels allows flux of ions and molecules through these channels, regulating various functions, such as cell adhesion, spreading, migration, proliferation, and differentiation. As an example, it has been shown that fluid flow-induced shear stress causes an increase in intracellular Ca^2+^ and GAG production in isolated *rabbit* meniscal cells (116), but the exact receptors responsible for sensing these signals and transporting the Ca^2+^ into the meniscal cells have yet to be identified (12). Candidates could be a family of transient receptor potential (TRP) channels that can be activated by various mechanical and chemical stimuli. TRP activation is known to increase intracellular Ca^2+^ and induce various cell responses (117). For example, TRPV4 activity is important for maintaining knee joint health of *mice* (118), and TRPV5 expression appears to be regulated by mechanical loading in articular cartilage of *rats* (119). However, exact mechanisms of channel-mediated communication between chondrocytes and their microenvironment are explored neither in human nor in dog. Uncovering the role of these receptors in mechanosensing of cartilaginous tissues, including meniscus, can improve our understanding of cell–ECM communication thus facilitating novel preventative and therapeutic strategies for meniscal pathology and the development of functional tissue-engineered meniscal implants.

### Oxidative Damage

Oxidative damage of macromolecules is mediated by reactive oxygen species (ROS), among which superoxide anion radical $\left({\text{O}}_{2}^{-}\right)$, hydroxyl radical (OH^⋅^), and nitric oxide (NO) are most prominent. ROS formation is a consequence of aerobic metabolism, normal aging processes, and exogenous stress. Cellular responses to ROS depend on the ROS concentration and the duration of ROS exposure. Although balanced ROS at nanomolar concentrations act as signaling molecules in various physiological functions, extensive ROS formation damages both cells and ECM (120). To ensure a properly maintained redox balance, organisms require a complex, coordinated network of antioxidants that control ROS production. Cellular and plasma antioxidants, such as superoxide dismutase, catalase, and glutathione peroxidase, counteract the formation of ROS. However, their activity can decrease with age and in degenerated tissues, as shown in human (121, 122). Additional radical scavengers, namely vitamins C and E, carotenoids, and flavonoids, can be obtained through dietary sources, but the delivery of these antioxidants into avascular tissues such as meniscus can be limited (123).

In contrast to many other cell types, the long lifetime of chondrocytes and fibrochondrocytes makes them particularly susceptible to the accumulation of oxidative damage from both aging and extrinsic stress (25). As shown in *human* studies, oxidative damage accelerates cell senescence and causes irreversible cell cycle arrest. The resulting senescence-associated secretory phenotype is characterized by active release of pro-inflammatory cytokines and an altered microenvironment, causing tissue fibrosis and loss of function (124–126). It has been confirmed that oxidative stress induces genomic instability and dysfunction of chondrocytes in human OA cartilage explants, suggesting that ROS are involved in the development of OA (127). Oxidative stress also activates production of nociceptive prostaglandins (PG) and causes time-dependent cell death of primary canine CCL cells. As elevated oxidative stress markers can be found in damaged human menisci (86, 95), it is likely that they also occur in degenerative and damaged canine menisci and play active roles in the development of pain. Recently, an *in vitro* study showed that *canine* chondrocytes are responsive to oxidative stress inducer hydrogen peroxide and its detrimental effects can be reduced by antioxidants (128). Furthermore, one can hypothesize that oxidative stress-induced damage positively correlates with degenerative state and/or patient’s age.

Oxidative stress also accelerates accumulation of damaging advanced glycation end products (AGEs) both inside and outside the cells. AGEs are modified proteins or lipids created by non-enzymatic glycation, due to the presence of reducing sugars (e.g., glucose). AGEs form irreversible cross-links in long-lived proteins of the ECM, preventing their repair and turnover (129). Accumulation of AGEs correlates with aging and development of knee OA both in *humans* and *dogs* (93, 130). In the canine CCL transection model, AGEs cause collagen damage and release of proteoglycans from CCL, impair its repair capacity, and reduce proteoglycan synthesis (131). Apart from the extracellular effects, AGEs can increase inflammatory responses of human articular and meniscal cells isolated from OA knees, by promoting the expression of cyclooxygenase-2 (COX-2) and the release of PG E2, MMP1, MMP3, MMP13, and TNF-α (87, 132). Intracellular accumulation of AGEs also induces apoptosis of chondrocytes *via* endoplasmic reticulum-related pathways (133). Deleterious effects of AGEs on the progression of human OA can be attenuated by inhibition of the receptor for advanced glycation end products (RAGE) (132, 134, 135). It has been confirmed that RAGE regulates a number of physiological and pathophysiological processes and RAGE-dependent signal transduction leads to the development of several human diseases (136). As elevated protein and mRNA expressions of RAGE were found in SF of human patients with knee OA (94), AGE–RAGE signaling appears to be a promising therapeutic target (137). No studies on AGE–RAGE in degenerative stifles of canine patients are available to assess the relevance of this therapeutic target in dogs.

It has been shown that nerve damage and resulting neuropathic pain are closely linked with the increased ROS levels (138). Degenerated cartilage from *human* OA patients contains elevated oxidative stress markers such as inducible nitric oxide synthase (iNOS) and nitrotyrosine, when compared with healthy samples (127, 139). Both iNOS and its metabolite NO were shown to intensify the sensation in neuropathic pain (140–142). Overproduction of NO was also found in SF isolated from *dogs* that underwent impact injury to the femoral condyle (97). In addition, NO can be involved in inflammatory responses that follow CCL damage in canine models (143). The importance of oxidative stress contributions to knee and stifle pain have not yet been well defined. Nevertheless, it has been shown that the MAP kinase pathways, which include ERK, JNK, and p38, can be involved (25, 143, 144). Molecules that interfere with oxidative stress, either by radical scavenging or by activation of cellular defense mechanisms, can be good therapeutic candidates to prevent ROS-related damage of musculoskeletal tissues. In preclinical tests, ROS scavengers were shown to reduce PG release and prevent oxidative stress-induced death of chondrocytes, suggesting that balancing production of ROS in damaged knees can slow down the progression of OA (145).

### The Role of the SF

Molecules released within degenerative or damaged knee/stifle joint spread through the SF, stimulating nerve endings and activating pain. In addition, degenerative processes influence hyaluronic acid (HA) quantity, quality, and residence time. HA, one of the main components of the SF, can be degraded by hyaluronidases, especially HYAL2, resulting in reduced joint lubrication, as shown in human studies. Expression of HYALs increases in synovium of *human* OA patients, while the expression of HA synthases is reduced (146). In addition to reduced joint lubrication, fragmented HA can have pro-inflammatory effects (147). Overproduction of ROS, e.g., by mechanical overloading, also accelerates HA fragmentation and consequently reduces joint lubrication (96, 148). Importantly, efficiency of HA-based treatments is decreased by ROS produced in damaged knees (149), underscoring the importance of oxidative stress in knee pathology. Fragmented HA has been found in OA knee joints both in human and *canine* patients and is correlated with lower load-bearing capacity (150, 151). It is likely that oxidative stress caused by meniscal pathology also negatively influences HA in dogs.

## Clinical Significance of Meniscal Pathology

Although a number of dysregulated molecular pathways have been implicated in pain both in human knees and canine stifles, pain mechanisms associated with damaged menisci are largely unknown. Two types of pain can occur: (1) neuropathic pain, resulting from neural damage, and (2) nociceptive pain, caused by a neural sensitization due to a tissue damage (152). Joint capsule, synovium, ligaments, and menisci are innervated by sensory nerves, which can be sensitized by various chemicals. Cytokines act as nociceptive triggers and also induce the expression of other potentially nociceptive molecules and direct pain mediators, such as NO and PG E (88). Biosynthesis of PG E2, mediated by COX-2, significantly increases in degenerative and injured human tissues, magnifying nociceptive and inflammatory responses (153). Neuropeptides such as substance P and nerve growth factor (NGF) are located in the sensory nerve endings, and their expression and release are associated with OA in humans (82, 154). In addition, neuropeptides can be produced by chondrocytes (155). NGF has been recognized as an important mediator of chronic pain and as a marker of inflammation (156, 157). NGF binds to two different receptors on the nociceptor neurons: (1) low-affinity 75-kDa neurotrophin receptors (p75NTR), which bind all neurotrophins, and (2) tropomyosin-related tyrosin kinase A (TrkA), which binds NGF more selectively than the other neurotrophins. NGF-activated p75NTR regulates a wide range of functions, including axonal growth, apoptosis, and myelination (158). NGF signaling through TrkA mediates the nociceptive functions of the sensory neurons through enhanced expression of substance P, calcitonin gene-related peptide, and transient receptor potential vanilloid 1 (156, 159). Therefore, NGF can be involved in both, pathological nerve ingrowth and nerve sensitization in painful human joints. As enhanced concentrations of NGF were also found in canine SF and correlated with chronic lameness, similar processes are likely involved in painful OA of both species (83). However, it is unclear whether the damaged meniscus plays an active role in NGF release.

Apart from transmission of pain signals, neuropeptides are involved in pathological neo-innervation of degenerative joints. By-products of cell metabolism, namely lactic acid, can also be noxious through dysregulation of acid sensing ion channels (160). Free radicals such as NO are involved in both inflammation and nerve damage of human cartilaginous tissues (141, 161). Despite the clinical importance of both neuropathic and nociceptive pain, mechanisms underlying pain in knee and stifle pathologies are not fully understood (3).

Current and prospective treatments for meniscal pathology are described in the Data Sheet S1 in Supplementary Material.

## Conclusion

Dogs with spontaneously occurring meniscal pathology have the potential of being an excellent model for studying mechanisms of degeneration and for testing new treatment strategies for both dogs and humans. A naturally occurring model of degeneration and injury may be appropriate especially for biologic-based and tissue engineering approaches, where pre-existing inflammatory and catabolic responses largely influence treatment outcomes. Although the anatomy, physiology, and pathology of the human and canine meniscus are similar, a detailed analysis of the known signaling pathways involved in spontaneous meniscus pathology in dogs is lacking. Due to lack of knowledge, it is currently not possible to fully assess the relevance of dogs with spontaneous meniscal pathology as a translational model. To evaluate the relevance of this model, more basic research on canine meniscal biology and pathophysiology is needed in the future.

## Author Contributions

All authors contributed to the manuscript, have read, and approved the final version. OK carried out the literature review, drafted the manuscript, and conceived funding. LS contributed to the manuscript draft. KW-K critically revised the manuscript. JC critically revised the manuscript. AP contributed to the manuscript draft and conceived funding.

## Conflict of Interest Statement

The authors declare that the research was conducted in the absence of any commercial or financial relationships that could be construed as a potential conflict of interest.
