# Supplementary material for: The Pathobiology of the Meniscus: A Comparison Between the Human and Dog
Source: Front Vet Sci. 2018 Apr 16;5:73. doi: 10.3389/fvets.2018.00073 (PMC5911564; doi:10.3389/fvets.2018.00073)
Supplement: Supplementary file 1 [file Data_Sheet_1.docx]

# 9. Supplement: Treatments for meniscal pathology

Despite the fast development in this area, more progress is needed to translate biological repair approaches into routine clinical application for meniscal tissue engineering. The use of canine patients with spontaneous meniscal pathology for testing tissue engineering-based therapies may advance translation of these treatments.

## 9.1 Pharmacotherapy

Pharmacologic treatment options available for pain relief in patients with knee pain include topical therapy, intra-articular therapy and oral analgesic therapy. A complete discussion on the different analgesic treatments has been provided elsewhere ^1^. The currently used pharmacologic treatments can be successful in relieving symptoms of pain in both acute and chronic cases. However, prolonged use carries the risks for the patients and does not prevent the progression of OA.

***Hyaluronic acid.*** Hyaluronic acid (HA) is a widely studied glycosaminoglycan with promising therapeutic effects on human and canine OA. In the canine model of meniscal release, intra-articular injections of HA resulted in clinically significant improvements with respect to pain and knee function, when compared to pre-treatment values and saline controls for 4–6 months after treatment. However, HA injections did not prevent development of OA ^2^. Intra-articular HA injections were also shown to reduce synovial inflammation, degradation of extracellular matrix, and limit pain ^3^. However, success of HA injection therapy can be significantly decreased by the presence of ROS in the joint, as free radicals, excessively produces by damaged cells, cause HA fragmentation ^4^. Small Mw HA fragments can in turn induce inflammatory and stress responses within the joint, further promoting OA ^5^. These results suggest that oxidative stress-modifying agents administered simultaneously with HA can possibly increase success of HA therapy. Based on the similarities in pre-existing inflammatory and oxidative stress responses between human and canine patients, dogs with spontaneous meniscal damage can be a good model to test such combination treatments. Currently, the American Academy of Orthopaedic Surgeons does not support use of HA in human knees

## 9.2 Surgery

Meniscal lesions require surgical intervention in approximately 85% of the cases in people, which is a relatively high percentage compared to other injuries of the knee joint ^6^. The goals of surgical intervention for meniscal tears are to relieve mechanical symptoms and pain, facilitate normal daily living activities, and mitigate progression of osteoarthritis of the knee ^7^. Surgical options for meniscal lesions include partial or total meniscectomy, meniscal repair or meniscal replacement. Treatment decision-making depends on several factors including the type and location of tear, age and level of activity of the patient and presence of co-morbidities and/or osteoarthritis. Although surgical treatment of meniscal pathology is still prevalent, a conservative approach is gaining increased acceptance especially when managing degenerative meniscal tears in lower-demand patients. Several randomized clinical trials showed no improved outcomes following arthroscopic partial meniscectomy compared to non-operative treatment in these types of cases ^8-10^ . A recent publication reported a consensus statement endorsed by the European Society of Sports Traumatology, Knee Surgery, Arthroscopy to facilitate clinical decision-making. A total of 84 surgeons and scientists from 22 European countries critically reviewed the literature and concluded that arthroscopic partial meniscectomy should not be recommended as first line treatment for degenerative meniscal tears. The main reason is that the patients may experience pain for other reasons such as early onset of osteoarthritis.

***Meniscal resection.*** Most meniscal injuries in both people and dogs are treated by partial resection of the loose or frayed fragments, defined as partial meniscectomy ^7; 11^. The effect of partial meniscectomy on the biomechanics of the joint has been studied in both people and dogs, showing that removal of meniscal tissue alters cartilage pressure and leads to osteoarthritis. Experimental studies in dogs have shown that the magnitude of cartilage degeneration depends on the extent of meniscal resection ^12^. For this reason, preservation of as much functional meniscal tissue as is possible is always recommended.

***Meniscal repair.*** With respect to meniscal repair, healing after repair is only likely in select tears and patients, primarily based on vascular supply to the meniscus and patient age. Meniscal tears found in the inner avascular region, which functions under a highly demanding mechani­cal environment, are considered to be a considerable challenge to healing ^13^. Tear morphologies such as flaps, radial tears, and degenerative tears are generally not repaired ^11; 14^. Tears in younger patients and those repaired concurrent to ACL reconstruction are more likely to heal. Several repair techniques facilitating suture alone or repair devices have been described in the peer-reviewed literature ^7^. Newer generation repair devices allow all-arthroscopic meniscal repair using sliding knots that can be tensioned by the surgeon for secure tear repair ^7^. Meniscal horn tears may be repaired using either suture anchors or transosseous suturing techniques. ^7^. Meniscal repair is rarely performed in dogs.

***Meniscal replacement***. When repair or preservation of functional meniscus is not feasible, replacement with meniscal allograft or synthetic implants can be considered. ^15^. In people, meniscal allograft transplantation has become an accepted management option for select symptomatic patients who have undergone a subtotal or total meniscectomy: fair to excellent functional results after meniscal allograft transplantation have been reported in 75-90% of treated patients (reported follow-up times: 3-14 years), with a clinical survivorship 10 years postoperatively at an estimated 70% for medial and lateral allografts ^7; 16^. Patients who develop symptoms of pain and swelling following meniscectomy are the typical candidates for this procedure. Contraindications for allograft implantation are advanced arthrosis, obesity, synovial disease, inflammatory arthritis, significant osteoarthritis and previous joint infection ^16^. In addition, a stable knee and correct axial alignment should be ensured. Drawbacks include the limited number of available grafts, cost, graft sizing, effects of sterilization and preservation on biomechanical strength of the graft, and the risk of disease transmission ^15; 16^. Meniscal allograft transplantation has been reported in dogs.

Synthetic meniscal scaffolds are emerging as a potential alternative to meniscal allograft transplantation. The goal of resorbable meniscal scaffolds is to allow in-growth of meniscal tissue and thereby create a regenerated meniscus over time formed by host tissue ^16^. Several synthetic replacements are currently under clinical investigation ^7^. Several other implants that have been tested in animal studies include implants derived from porcine small intestinal submucosa ^17^, polycaprolactone and hyaluronan-derived polymer reinforced with polylactic acid fibers or polyethylene terephthalate net ^18^, kevlar reinforced polycarbonate-urethane implants ^19^, and ultrahigh-molecular-weight polyethylene fiber reinforced polyvinyl-alcohol hydrogel implants ^7^. Materials that possess various attractive characteristics and behaviors for meniscal repair have been summarized in recent literature ^20-22^. Current challenges of meniscal replacement include the fixation (particularly of total meniscal implants), the material properties and surface characteristics.

## 9.3 Biological repair

***Molecular antagonists***. Activation of inflammatory and oxidative stress-related pathways in degeneration of cartilaginous tissues is associated with disease progression and pain ^23; 24^. Therefore, inflammation and oxidative stress are important therapeutic targets. It has been shown that the detrimental effects of pro-inflammatory cytokines IL-1 and TNF-α on porcine meniscal repair *in vitro* can be blocked by the administration of IL-1 receptor antagonist and anti-TNF monoclonal antibody, suggesting that specific molecular antagonists can become promising therapeutics ^25^. Similar study on human or canine menisci is not yet available. Antioxidants including N-acetyl-cysteine or vitamin E were shown to provide benefits in the management of human knee OA ^26^, possibly by reducing chondrocyte apoptosis ^27; 28^. However, recent studies have revealed that inhibition of a single molecule or pathway may not be sufficient for the successful treatment of knee pathologies, as multiple catabolic factors are involved in their pathogenesis and should to be simultaneously targeted ^28^. For testing combinations of molecular antagonists, an animal model with pre-existing pathophysiological/degenerative processes will be ideal.

***Growth factors.*** Growth factors (GF) are polypeptides used as ECM-stimulating agents. Growth factors such as bone morphogenetic proteins (BMPs), insulin growth factor (IGF-1), tumor growth factor (TGF-β), epidermal growth factor (EGF), fibroblast growth factor (FGF) and growth differentiation factor-5 (GDF-5), were shown to induce the formation of new ECM and suppress activity of catabolic enzymes, both in human chondrocytes and meniscal cells ^29; 30^. The major drawback of exogenous GF is their short half-life (hours to days), resulting in only transient effects and the need for repeated injections. Another drawback can be their high costs. GF can induce marked cellular responses to improve meniscal repair, but optimal doses of GF for clinical use still need to be determined. For this purpose, dogs with spontaneous meniscal pathologies can also be appropriate.

***Platelet rich plasma***. Autologous platelet rich plasma (PRP) contains mixture of activated platelets releasing GF (PDGF, FGF, EGF, IGF-1) and healing-promoting cytokines. PRP, injected directly in the damaged tissue, is thought to stimulate healing and shift the catabolism of muskulokeletal tissues towards the anabolic state ^31^. In a canine CCL transection model with meniscal release, multiple intra-articular injections of PRP during 6 months reduced pain and improved limb function, when compared to saline-treated dogs. However, these injections did not reduce the risk of progressive OA ^32^. Despite promising results, the overall success of PRP-based treatments in meniscal and cartilage healing has been only modest so far ^33^, likely due to variable composition of PRP, lack of scaffold, and catabolic environment reducing half-life of PRP components. The composition of PRP requires standardization and evaluation through animal testing to advance clinical translation of this strategy ^34^.

***Meniscal fibrochondrocytes and synoviocytes.*** Autologous meniscal cells and other cell types such as chondrocytes and synoviocytes have been used to restore meniscal function both in human and dog ^35^. However, these cells are usually obtained from surgical specimens, thus they can be affected by the degenerative process. In addition, meniscal cells suffer from poor proliferation rate and the loss of native phenotypic features during the expansion in monolayer. Nevertheless, these drawbacks can be overcome by specific culture conditions and use of scaffolds ^35-37^. Regenerative potential of autologous synoviocytes and meniscal fibrochondrocytes isolated from canine patients with naturally occurring meniscal damage was tested using fibrocartilage scaffolds. Both synoviocytes and meniscal fibrochondrocytes seeded in these scaffolds produced ECM, but the viability of meniscal fibrochondrocytes was reduced, possibly due to their diseased state ^38^. Interestingly, canine synoviocytes derived from OA joints produced meniscal ECM at levels similar to those of normal synoviocytes ^39^. These canine studies demonstrated that synoviocytes isolated from patients with meniscal damage can be a potential source for meniscus tissue engineering.

***Stem cells.*** Stem cells are able to self-renew, maintaining their undifferentiated phenotype in multiple subcultures. Appropriate stimuli induce differentiation of stem cells towards specific tissues. Additionally, a paracrine effect of stem cells has been described, suggesting that they may act as mediators to stimulate healing and decrease inflammation rather than directly regenerating tissue ^40^. Both undifferentiated and differentiated adult stem cells, such as bone marrow-derived mesenchymal stem cells (BMSCs) of adipose tissue-derived stem cells (ASCs), were used for meniscal tissue engineering in human and dog ^41^. In addition, direct injection of mesenchymal stem cells (MSCs) into the knee joints with damaged menisci has been assessed in human clinical trials ^42^. Application of MSCs was shown to be safe, temporarily increase meniscus volume and reduced pain, thus indicating promise for meniscal healing. In canine model of scalpel-induced meniscal tears, BMSCs enhanced meniscal wound healing by increasing angiogenesis, chondrogenesis and collagen deposition in the damaged menisci ^43^. In canine patients with CCL ruptures (accompanied by secondary meniscal damage in 27% of cases), culture-expanded autologous BMSCs injected intravenously or locally reduced serum and synovial fluid inflammation over an 8-week period, but effects on pain and limb function were not reported ^44^. Although promising outcomes were found in *in vivo* studies, no fully conclusive data on the long-term efficiency of stem cell therapy for meniscus repair exist to date. Importantly, survival of exogenous stem cells depends on surrounding microenvironment, which can be unfavorable in case of highly catabolic and inflamed tissues. Nevertheless, the use of stem cells in humans can still be problematic due to issues with batch consistency as well as product stability, safety and efficacy through pre-clinical and clinical studies ^45^.

# 8. References

1. Walker-Bone K, Javaid K, Arden N, et al. 2000. Regular review: medical management of osteoarthritis. Bmj 321:936-940.

2. Pashuck TD, Kuroki K, Cook CR, et al. 2016. Hyaluronic acid versus saline intra-articular injections for amelioration of chronic knee osteoarthritis: A canine model. Journal of Orthopaedic Research 34:1772-1779.

3. Muir P, Schwartz Z, Malek S, et al. 2011. Contralateral cruciate survival in dogs with unilateral non-contact cranial cruciate ligament rupture. PloS one 6:e25331.

4. Conrozier T, Mathieu P, Rinaudo M. 2014. Mannitol Preserves the Viscoelastic Properties of Hyaluronic Acid in an In Vitro Model of Oxidative Stress. Rheumatol Ther 1:45-54.

5. Rayahin JE, Buhrman JS, Zhang Y, et al. 2015. High and Low Molecular Weight Hyaluronic Acid Differentially Influence Macrophage Activation. Acs Biomater Sci Eng 1:481-493.

6. Majewski M, Susanne H, Klaus S. 2006. Epidemiology of athletic knee injuries: A 10-year study. Knee 13:184-188.

7. Fox AJ, Wanivenhaus F, Burge AJ, et al. 2015. The human meniscus: a review of anatomy, function, injury, and advances in treatment. Clin Anat 28:269-287.

8. Herrlin S, Hallander M, Wange P, et al. 2007. Arthroscopic or conservative treatment of degenerative medial meniscal tears: a prospective randomised trial. Knee Surg Sports Traumatol Arthrosc 15:393-401.

9. Kirkley A, Birmingham TB, Litchfield RB, et al. 2008. A randomized trial of arthroscopic surgery for osteoarthritis of the knee. The New England journal of medicine 359:1097-1107.

10. Herrlin SV, Wange PO, Lapidus G, et al. 2013. Is arthroscopic surgery beneficial in treating non-traumatic, degenerative medial meniscal tears? A five year follow-up. Knee Surg Sports Traumatol Arthrosc 21:358-364.

11. Pozzi A, Kowaleski MP, Apelt D, et al. 2006. Effect of medial meniscal release on tibial translation after tibial plateau leveling osteotomy. Veterinary surgery : VS 35:486-494.

12. Cox JS, Nye CE, Schaefer WW, et al. 1975. The degenerative effects of partial and total resection of the medial meniscus in dogs' knees. Clinical orthopaedics and related research:178-183.

13. Englund M, Roemer FW, Hayashi D, et al. 2012. Meniscus pathology, osteoarthritis and the treatment controversy. Nature reviews Rheumatology 8:412-419.

14. Laible C, Stein DA, Kiridly DN. 2013. Meniscal repair. The Journal of the American Academy of Orthopaedic Surgeons 21:204-213.

15. Brophy RH, Matava MJ. 2012. Surgical options for meniscal replacement. The Journal of the American Academy of Orthopaedic Surgeons 20:265-272.

16. Verdonk R, Volpi P, Verdonk P, et al. 2013. Indications and limits of meniscal allografts. Injury 44 Suppl 1:S21-27.

17. Cook JL, Fox DB, Malaviya P, et al. 2006. Long-term outcome for large meniscal defects treated with small intestinal submucosa in a dog model. The American journal of sports medicine 34:32-42.

18. Chiari C, Koller U, Dorotka R, et al. 2006. A tissue engineering approach to meniscus regeneration in a sheep model. Osteoarthritis and cartilage / OARS, Osteoarthritis Research Society 14:1056-1065.

19. Zur G, Linder-Ganz E, Elsner JJ, et al. 2011. Chondroprotective effects of a polycarbonate-urethane meniscal implant: histopathological results in a sheep model. Knee Surg Sports Traumatol Arthrosc 19:255-263.

20. Longo UG, Loppini M, Forriol F, et al. 2012. Advances in meniscal tissue engineering. Stem Cells Int 2012:420346.

21. Mauck RL, Burdick JA. 2015. From repair to regeneration: biomaterials to reprogram the meniscus wound microenvironment. Annals of biomedical engineering 43:529-542.

22. Vrancken AC, Buma P, van Tienen TG. 2013. Synthetic meniscus replacement: a review. International orthopaedics 37:291-299.

23. Wilusz RE, Weinberg JB, Guilak F, et al. 2008. Inhibition of integrative repair of the meniscus following acute exposure to interleukin-1 in vitro. Journal of orthopaedic research : official publication of the Orthopaedic Research Society 26:504-512.

24. Ziskoven C, Jager M, Zilkens C, et al. 2010. Oxidative stress in secondary osteoarthritis: from cartilage destruction to clinical presentation? Orthop Rev (Pavia) 2:e23.

25. McNulty AL, Moutos FT, Weinberg JB, et al. 2007. Enhanced integrative repair of the porcine meniscus in vitro by inhibition of interleukin-1 or tumor necrosis factor alpha. Arthritis and rheumatism 56:3033-3042.

26. Grover AK, Samson SE. 2016. Benefits of antioxidant supplements for knee osteoarthritis: rationale and reality. Nutr J 15:1.

27. Martin JA, McCabe D, Walter M, et al. 2009. N-acetylcysteine inhibits post-impact chondrocyte death in osteochondral explants. The Journal of bone and joint surgery American volume 91:1890-1897.

28. Kramer WC, Hendricks KJ, Wang J. 2011. Pathogenetic mechanisms of posttraumatic osteoarthritis: opportunities for early intervention. International journal of clinical and experimental medicine 4:285-298.

29. McNulty AL, Guilak F. 2008. Integrative repair of the meniscus: lessons from in vitro studies. Biorheology 45:487-500.

30. Hui W, Rowan AD, Cawston T. 2001. Modulation of the expression of matrix metalloproteinase and tissue inhibitors of metalloproteinases by TGF-beta1 and IGF-1 in primary human articular and bovine nasal chondrocytes stimulated with TNF-alpha. Cytokine 16:31-35.

31. Ishida K, Kuroda R, Miwa M, et al. 2007. The regenerative effects of platelet-rich plasma on meniscal cells in vitro and its in vivo application with biodegradable gelatin hydrogel. Tissue engineering 13:1103-1112.

32. Cook JL, Smith PA, Bozynski CC, et al. 2016. Multiple injections of leukoreduced platelet rich plasma reduce pain and functional impairment in a canine model of ACL and meniscal deficiency. Journal of Orthopaedic Research 34:607-615.

33. Griffin JW, Hadeed MM, Werner BC, et al. 2015. Platelet-rich Plasma in Meniscal Repair: Does Augmentation Improve Surgical Outcomes? Clinical orthopaedics and related research 473:1665-1672.

34. Carr BJ, Canapp SO, Jr., Mason DR, et al. 2015. Canine Platelet-Rich Plasma Systems: A Prospective Analysis. Front Vet Sci 2:73.

35. Niu W, Guo WM, Han SF, et al. 2016. Cell-Based Strategies for Meniscus Tissue Engineering. Stem Cells Int.

36. Gunja NJ, Athanasiou KA. 2007. Passage and reversal effects on gene expression of bovine meniscal fibrochondrocytes. Arthritis research & therapy 9.

37. Tan GK, Dinnes DLM, Myers PT, et al. 2011. Effects of biomimetic surfaces and oxygen tension on redifferentiation of passaged human fibrochondrocytes in 2D and 3D cultures. Biomaterials 32:5600-5614.

38. Ballard GA, Warnock JJ, Bobe G, et al. 2014. Comparison of meniscal fibrochondrocyte and synoviocyte bioscaffolds toward meniscal tissue engineering in the dog. Res Vet Sci 97:400-408.

39. Warnock JJ, Bobe G, Duesterdieck-Zellmer KF. 2014. Fibrochondrogenic potential of synoviocytes from osteoarthritic and normal joints cultured as tensioned bioscaffolds for meniscal tissue engineering in dogs. Peerj 2.

40. Wang MJ, Yuan ZG, Ma N, et al. 2017. Advances and Prospects in Stem Cells for Cartilage Regeneration. Stem Cells Int.

41. Yu HN, Adesida AB, Jomha NM. 2015. Meniscus repair using mesenchymal stem cells - a comprehensive review. Stem cell research & therapy 6.

42. Vangsness CT, Farr J, Boyd J, et al. 2014. Adult Human Mesenchymal Stem Cells Delivered via Intra-Articular Injection to the Knee Following Partial Medial Meniscectomy A Randomized, Double-Blind, Controlled Study. Journal of Bone and Joint Surgery-American Volume 96a:90-98.

43. Abdel-Hamid M, Hussein MR, Ahmad AF, et al. 2005. Enhancement of the repair of meniscal wounds in the red-white zone (middle third) by the injection of bone marrow cells in canine animal model. Int J Exp Pathol 86:117-123.

44. Muir P, Hans EC, Racette M, et al. 2016. Autologous Bone Marrow-Derived Mesenchymal Stem Cells Modulate Molecular Markers of Inflammation in Dogs with Cruciate Ligament Rupture. PloS one 11.

45. George B. 2011. Regulations and guidelines governing stem cell based products: Clinical considerations. Perspect Clin Res 2:94-99.
